# Supplementary material for: Topical Application of Bio-Pulsed Avian MSC-Derived Extracellular Vesicles Enhances Hair Regrowth and Skin Rejuvenation: Evidence from Clinical Evaluation and miRNA Profiling
Source: Curr Issues Mol Biol. 2025 Jul 11;47(7):539. doi: 10.3390/cimb47070539 (PMC12293773; doi:10.3390/cimb47070539)
Supplement: Supplementary file 1 [file cimb-47-00539-s001.zip › cimb-3710882-supplementary.pdf]

Supplementary_Table_S1_Hair Questionnaire

Subjective Hair Evaluation Questionnaire

Participants completed this 5-point Likert scale questionnaire at Day 30 and Day 60 post-treatment. Participants responded using a 5-point Likert scale: 5 = Very satisfied, 4 = Satisfied, 3 = Neutral, 2 = Dissatisfied, 1 = Very dissatisfied.

Name: ________________

Please select your age group: ☐ 30–45 years; ☐ 46–65years

Section A: Day 30 Evaluation

No.	Evaluation Item	Response (1–5)
A1	Reduced hair shedding	
A2	Increased hair volume	
A3	New hair growth	
A4	Strengthened hair roots	
A5	More active and healthier scalp	
A6	Reduced scalp sensitivity and itchiness	
A7	Reduced scalp oiliness	
A8	Hair appears fuller and more elastic	

Section B: Day 60 Evaluation

No.	Evaluation Item	Response (1–5)
B1	Reduced hair shedding	
B2	Increased hair volume	
B3	New hair growth	
B4	Strengthened hair roots	
B5	More active and healthier scalp	
B6	Reduced scalp sensitivity and itchiness	
B7	Reduced scalp oiliness	
B8	Hair appears fuller and more elastic	

Supplementary_Table_S2_Skin Questionnaire

Subjective Skin Evaluation Questionnaire

This questionnaire was administered to participants to evaluate subjective skin improvement after 14 and 28 days of using the Bio-Pulsed Exosome Essence (ExoGiov®). Participants responded using a 5-point Likert scale: 5 = Very satisfied, 4 = Satisfied, 3 = Neutral, 2 = Dissatisfied, 1 = Very dissatisfied.

Name: ________________

Please select your age group: ☐ 30–45 years; ☐ 46–65years

Usage: Apply ExoGiov® Bio-Pulsed Exosome Essence to the face twice daily for 4 consecutive weeks.

Section A: After 14 Days of Use

No.	Evaluation Item	Response (1–5)
A1	Reduce facial wrinkles and fine lines	
A2	Improved skin firmness	
A3	Improved skin elasticity	
A4	Improve enlarged pores	
A5	Brighten skin tone	
A6	Improve skin gloss	
A7	Improve uneven skin tone	
A8	Reduce facial spots	
A9	Reduce sensitive erythema on face	
A10	Overall skin quality improvement	

Section B: After 28 Days of Use

No.	Evaluation Item	Response (1–5)
A1	Reduce facial wrinkles and fine lines	
A2	Improved skin firmness	
A3	Improved skin elasticity	
A4	Improve enlarged pores	
A5	Brighten skin tone	
A6	Improve skin gloss	
A7	Improve uneven skin tone	
A8	Reduce facial spots	
A9	Reduce sensitive erythema on face	
A10	Overall skin quality improvement	

Supplementary Table S3

Complete excipient composition and concentrations of ExoGiov® Bio-Pulsed Exosome formulations (Essence, Blue Ampoule, Silver Ampoule).

INCI Name	Function	Essence (%)	Blue Ampoule (%)	Silver Ampoule (%)
AQUA	Solvent	98.0000	95.1600	95.9160
AMMONIUM ACRYLOYLDIMETHYLTAURATE/VP COPOLYMER	Thickener	0.2000	-	-
AMMONIUM POLYACRYLOYLDIMETHYL TAURATE	Thickener	0.3920	-	-
t-BUTYL ALCOHOL	Stabilizer	0.0080	-	-
INOSITOL	Moisturizer	3.0000	-	-
CREATINE	Moisturizer	0.1500	-	-
GLYCERYL CAPRYLATE	Humectant	-	0.6000	0.6000
PROPYLENE GLYCOL	Humectant	5.2000	0.6000	0.6000
PENTYLENE GLYCOL	Humectant	-	0.6000	0.6000
PROPANEDIOL	Humectant	2.3000	3.0000	3.0000
CAPRYLHYDROXAMIC ACID	Preservative	-	0.6000	0.6000
CHLORPHENESIN	Preservative	0.2000	-	-
PHENOXYETHANOL	Preservative	0.3150	-	-
HYDROXYACETOPHENONE	Antioxidant	0.2000	0.4000	0.4000
SODIUM HYALURONATE	Humectant	0.1500	-	-
DISODIUM EDTA	Chelating Agent	0.1000	-	-
GLYCERETH-26	Moisturizer	1.0000	-	-
ALOE BARBADENSIS LEAF JUICE	Soothing Agent	2.9790	-	-
SODIUM BENZOATE	Preservative	0.1000	-	-
CITRIC ACID	pH Adjuster	0.0150	-	-
POTASSIUM SORBATE	Preservative	0.0060	-	-
Chicken Embryonic MSC-Derived Extracellular Vesicles (ExoGiov)	Active Ingredient	0.0168	0.2000	0.0840
L-ARGININE	Skin Conditioner	0.0250	-	-
